# Forecasting COVID-19 cases in US states using reconstructed incidence data

**DOI:** 10.1371/journal.pone.0353983

**Published:** 2026-08-24

**Authors:** Rebecca K. Nash, Sangeeta Bhatia, Jack Wardle, Anne Cori, Pierre Nouvellet

**Affiliations:** 1 MRC Centre for Global Infectious Disease Analysis, School of Public Health, Imperial College London, London, United Kingdom; 2 School of Life Sciences, University of Sussex, Brighton and Hove, United Kingdom; Shanxi University, CHINA

## Abstract

Branching process models are commonly used in infectious disease forecasting and often rely on daily incidence data, but their utility can be restricted if incidence is not reported daily or if reporting becomes less frequent during prolonged outbreaks. In this study, an Expectation Maximisation algorithm is used to reconstruct the daily incidence of COVID-19 cases from weekly case counts. Using data from 13 US states that maintained mostly daily reporting of COVID-19 cases from March 2020 to February 2022, we evaluate forecasting performance by comparing models using the true daily incidence with those using reconstructed daily incidence. Our results show that forecasts generated from reconstructed incidence perform equally well as those generated using true daily incidence. These findings demonstrate the viability of using reconstructed incidence data for real-time forecasting, which could be particularly useful in scenarios where maintaining daily reporting is unsustainable or in settings with limited surveillance capacity.

## Introduction

Infectious disease forecasts are a crucial element of outbreak response, allowing us to estimate the likely number of future cases, hospitalisations or deaths. These predictions allow public health agencies to implement timely interventions and allocate resources more effectively to mitigate the impact of disease spread.

One common type of forecasting model is the branching process model, which is often based on the renewal equation and relies on past daily incidence data, the latest estimate for the time-varying reproduction number ($R_{t}$ – the average number of cases caused by a primary case infected at time *t* of an outbreak, assuming that conditions remain the same after time *t*) and either the serial interval (SI) or generation time. Using these inputs, future incidence can be projected over a specified time window assuming that $R_{t}$ remains constant. Throughout the COVID-19 pandemic, caused by the SARS-CoV-2 virus, branching process models have been used to forecast future cases, hospitalisations and deaths to inform real-time response globally [1–3].

In practice, the incidence of many infectious diseases is not reported daily and may instead be reported irregularly or as weekly counts, which can be a barrier to the use of these models in such contexts [4]. For example, due to the protracted nature of the COVID-19 pandemic and the demand on resources, many countries, regions and public health agencies did not maintain daily reporting of COVID-19 incidence [5–8]. Although methods have been developed to reconstruct daily incidence from aggregated counts for the purpose of estimating transmissibility [9,10], the impact of using reconstructed incidence on the accuracy of forecasts is unknown. Understanding these implications is important not only for determining the viability of using reconstructed incidence for forecasting when daily incidence is unavailable, but also for informing the design of surveillance systems for future epidemics. To investigate this, we use the incidence of COVID-19 cases in the United States (US) as a case study.

**Fig 1:**
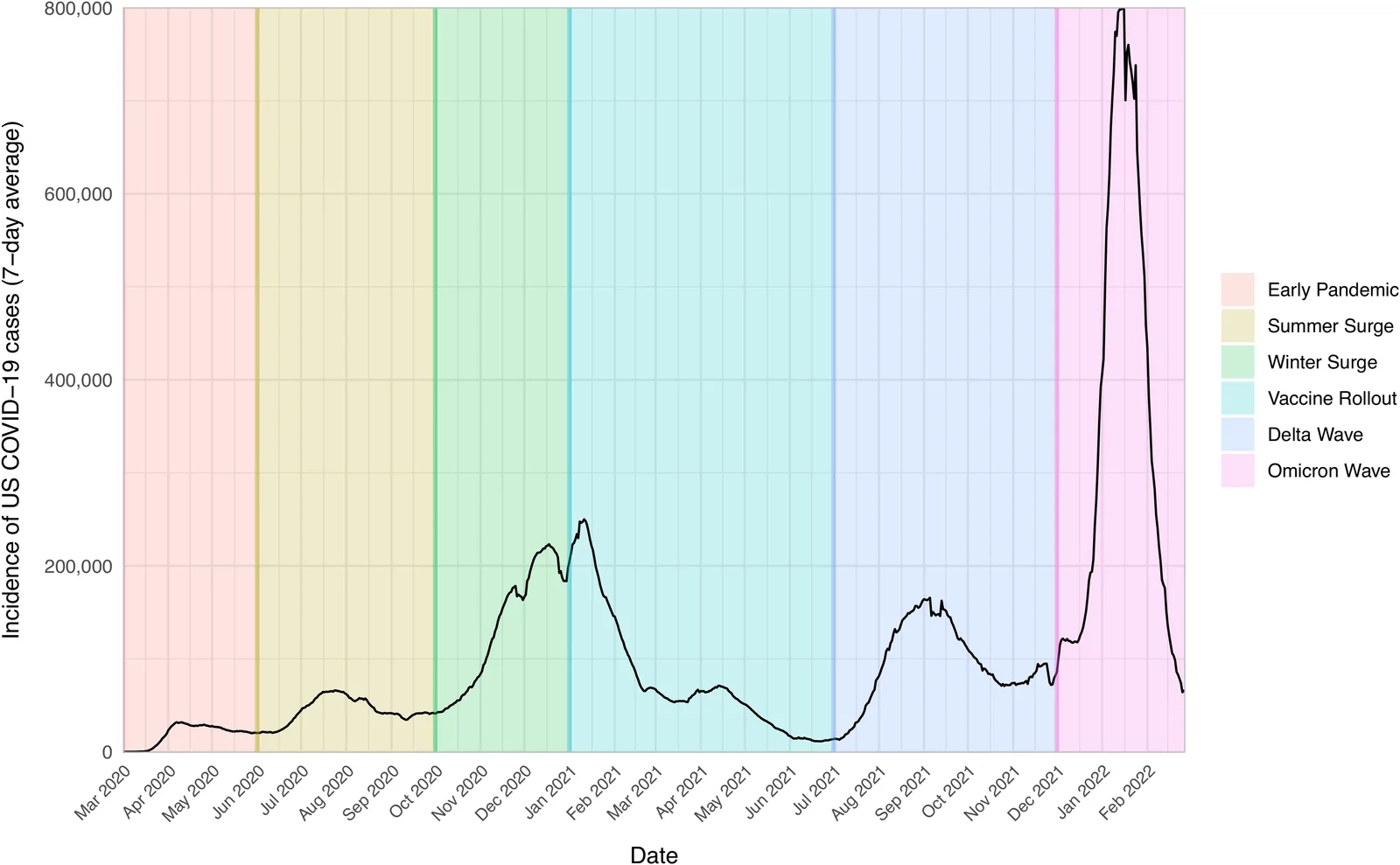
Incidence of COVID-19 cases in the US (7-day rolling average) from March 2020 to February 2022. Highlighted periods represent key phases of the pandemic, including: ‘Early Pandemic’ (March – May 2020), ‘Summer Surge’ (June – September 2020), ‘Winter Surge’ (October – December 2020), ‘Vaccine Rollout’ (January – June 2021), ‘Delta Wave’ (July – November 2021) and ‘Omicron Wave’ (December 2021 – February 2022).

In the US, the first laboratory-confirmed case of COVID-19 was reported in Washington state on 20th January 2020 following recent travel to Wuhan, China [11]. On 11th March 2020, the World Health Organisation officially declared COVID-19 a pandemic [12]. In the two years following this declaration (March 2020 – February 2022), the US experienced six distinct outbreak phases, which we have defined as the: ‘Early Pandemic’, ‘Summer Surge’, ‘Winter Surge’, ‘Vaccine Rollout’, ‘Delta Wave’ and ‘Omicron Wave’ (Fig 1).

While initial data collection for COVID-19 cases was largely standardised, the autonomy of individual states and overwhelmed healthcare systems led to variations in reporting frequencies and intervention strategies as the pandemic progressed [13,14]. Early in the pandemic, daily reporting was typically expected. However, as cases began to steadily decline during the ‘Vaccine Rollout’ phase (January – June 2021) many US states reduced the reporting frequency of cases and deaths, typically shifting to reporting 4–5 days per week [7,15].

Despite the reduction in reporting frequency across many US states, two waves of COVID-19 cases occurred: the ‘Delta Wave’ (July – November 2021) and the largest ‘Omicron Wave’ (December 2021 – February 2022), driven by the emergence of the highly transmissible, though less severe, Omicron variant [16]. From March 2022 onward, the policy approach to COVID-19 shifted toward endemicity across the US, focusing on long-term management strategies [17]. At this stage, the vast majority of US states had ceased daily reporting of cases and deaths.

In this study, we aim to use a real-world case study to assess whether daily reconstructed data can reliably support accurate forecasting and response planning in instances where daily reporting has ceased. Additionally, we assess whether maintaining daily reporting offers a significant advantage over the use of reconstructed data in terms of forecasting accuracy. To this end, we use COVID-19 case incidence from 13 US states that maintained mostly daily reporting as a case study. We seek to interrogate how the EM approach performs across US states and the epidemic phases of the COVID-19 pandemic and the extent to which reporting patterns influence the accuracy of results. We compare the forecasting performance of a branching process model using the original daily reported incidence and daily incidence reconstructed from a simulated weekly reporting scenario.

## Materials and methods

### COVID-19 data

Incidence of COVID-19 cases in the US were obtained from the COVID-19 Data Repository by the Center for Systems Science and Engineering at Johns Hopkins University [18,19]. The time series chosen encompasses two years of COVID-19 circulation in the US from March 2020 to February 2022. Case incidence data for 13 states were chosen for the analysis as they maintained mostly daily reporting throughout this time period. These states include California, Colorado, Delaware, Hawaii, Maryland, Missouri, New Jersey, New York, North Dakota, Ohio, Pennsylvania, Tennessee and Texas (accounting for around 45% of the US population [20]).

### Descriptive analysis

To identify reporting ‘noise’ (such as ‘weekend effects’ [9]) in case incidence data and examine how these patterns differ across states and outbreak phases (Fig 1), we conducted a descriptive analysis to identify days of the week with consistently higher or lower numbers of reported cases. We achieved this by calculating the percentage of weekly cases reported on each day and summarised these daily percentages across weeks using boxplots, which display the median, interquartile range, and overall spread of percentages (excluding outliers).

### Expectation Maximisation algorithm

For each included US state, we artificially aggregated the reported daily incidence data to weekly counts. Hereafter we refer to reported and reconstructed incidence data – these are the reported daily incidence and the daily incidence that has been reconstructed from weekly aggregated data, respectively. To reconstruct daily incidence from these weekly counts, we used an Expectation Maximisation (EM) algorithm [9]. The EM algorithm was initialised with a uniform distribution of cases across each week. The iterative process alternated between 1) an expectation step, where the time-varying reproduction number, $R_{t}$, was estimated for each aggregation window in turn using a chosen SI distribution (mean 4.8 days and standard deviation 2.7) [21]. This $R_{t}$ was subsequently translated into an exponential growth rate [22], which was used to distribute the total weekly cases across individual days; and 2) a maximisation step, where $R_{t}$ was re-estimated based on this updated daily distribution of cases. Iterations continued until convergence was reached.

To ensure that the integrity of the data was maintained, the reconstruction was constrained to ensure that the sum of reconstructed daily case counts match the reported weekly totals. The EM algorithm was implemented using the EpiEstim R package [23], and both the reported and reconstructed daily incidence data and the same SI distribution were supplied as inputs to the forecasting model.

As a supplementary analysis, we visually compared the reconstructed daily incidence data to the reported daily incidence and calculated the daily residuals (reported minus reconstructed case counts) to assess whether the reconstruction introduced systematic bias.

### Model

The renewal equation (Eq (1)) is used to forecast the future incidence of COVID-19 cases [23]. The model assumes that the incidence of new cases on day *t* (denoted $I_{t}$) can be represented by a Poisson process:


$$\begin{array}{c} I_{t}\sim \text{Pois}\left(R_{t}\sum_{s=1}^{t}I_{t-s}\omega_{s}\right) \end{array}$$ (1)

where $R_{t}$ is the time-varying reproduction number and the past incidence ($I_{t-s}$) is weighted by $\omega_{s}$, the probability mass function of the SI distribution (mean = 4.8 days, SD = 2.7 days [21]) [24,25].

To calibrate the model and smooth out ‘weekend effects’ in the data, $R_{t}$ was estimated using case incidence within a fixed 10-day calibration window prior to the projection date, where $R_{t}$ is assumed to remain constant [9,26]. Joint estimation of $R_{t}$ and the preceding incidence was performed via MCMC (10,000 iterations) using the ‘jointlyr’ package [27]. From the resulting distribution, 1,000 parameter sets were sampled, each producing 10 stochastic realisations to generate 10,000 trajectories per 28-day forecast [26].

Forecasts were generated weekly between 9 March 2020 and 27 February 2022, initiated on each day of the week to explore whether the starting day influenced forecast performance. Projections were excluded for states reporting zero cases in the previous week or suspected non-daily reporting (defined as more than 30 cases reported in a week with at least one day of zero incidence).

Performance was evaluated using the Continuous Ranked Probability Score (CRPS) on $\log_{10}(x+1)$ transformed counts to standardise for varying outbreak magnitudes across states [28]. CRPS is a measure of the absolute difference between the observed weekly cases in the forecast period and the forecast distribution. Therefore, the lower the CRPS value, the closer the forecast is to the true observed cases for that forecast week. CRPS calculations were implemented via the scoringutils and scoringRules packages [29,30] and compared between the included US states and the outbreak time periods as defined in Fig 1.

## Results

### Incidence reconstruction

The EM reconstruction of daily incidence data was compared to the raw daily reported counts (S1 Fig). Administrative noise, exhibited by extreme spikes and troughs in the reported daily incidence, was smoothed during the reconstruction. The magnitude of administrative noise varied across the included US states, but was most pronounced during the peaks of the Winter Surge, Delta Wave, and Omicron Wave. The daily residuals (reported minus reconstructed counts) consistently oscillated around zero, which suggests that the reconstruction did not introduce any systematic under- or over-estimation of case incidence.

### Reporting patterns by US state

**Fig 2:**
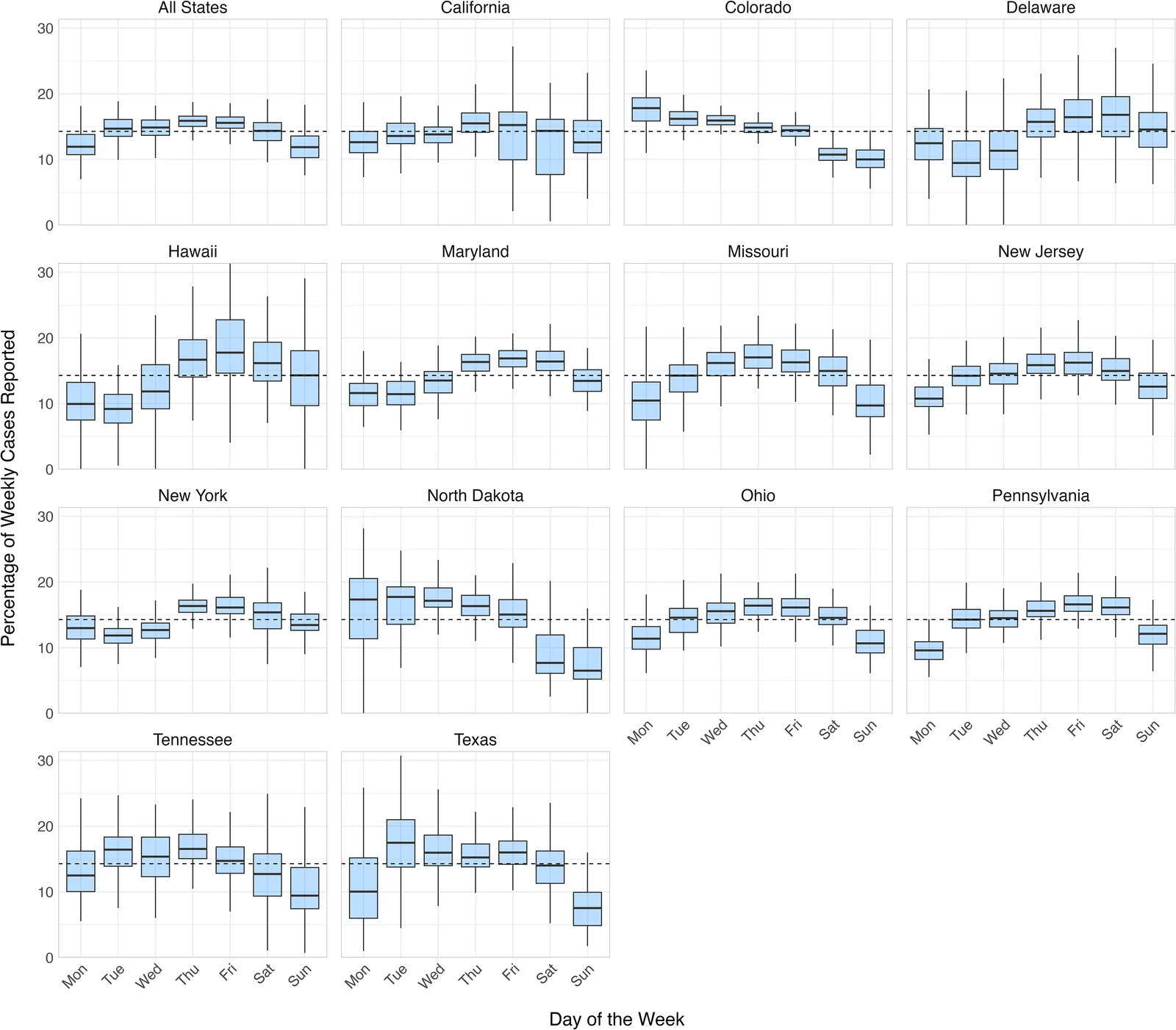
Relative reporting of COVID-19 cases by day of the week for each US state. Boxplots show the distribution of the percentage of weekly cases reported by day of the week, across weeks. Boxes represent the interquartile range (IQR), lines show medians, and whiskers extend to the most extreme values within 1.5 × IQR. Dashed lines represent the expected percentage if cases were reported equally on each day.

The relative reporting of US COVID-19 cases on each day of the week compared to the weekly total varied according to the US state (Fig 2). Aggregated across all included states, the median percentage of weekly reported cases was lower on Sundays and Mondays, with a peak midweek on Thursdays and Fridays. All 13 included states generally reported fewer cases on Sundays, Mondays or Tuesdays. Colorado and North Dakota displayed a typical ‘weekend-effect’ with lower reporting on weekends and high reporting at the start of the week. Most states, excluding Colorado, Delaware, North Dakota and Texas, had highest case counts on Thursdays or Fridays. The degree of week-to-week variability in daily reporting patterns differed between states. States such as Hawaii and Delaware showed more inconsistency in the percentage of weekly cases reported on different days of the week, whereas states such as Colorado and New York exhibited more consistent and stable reporting trends.

### Reporting patterns by outbreak phase

**Fig 3:**
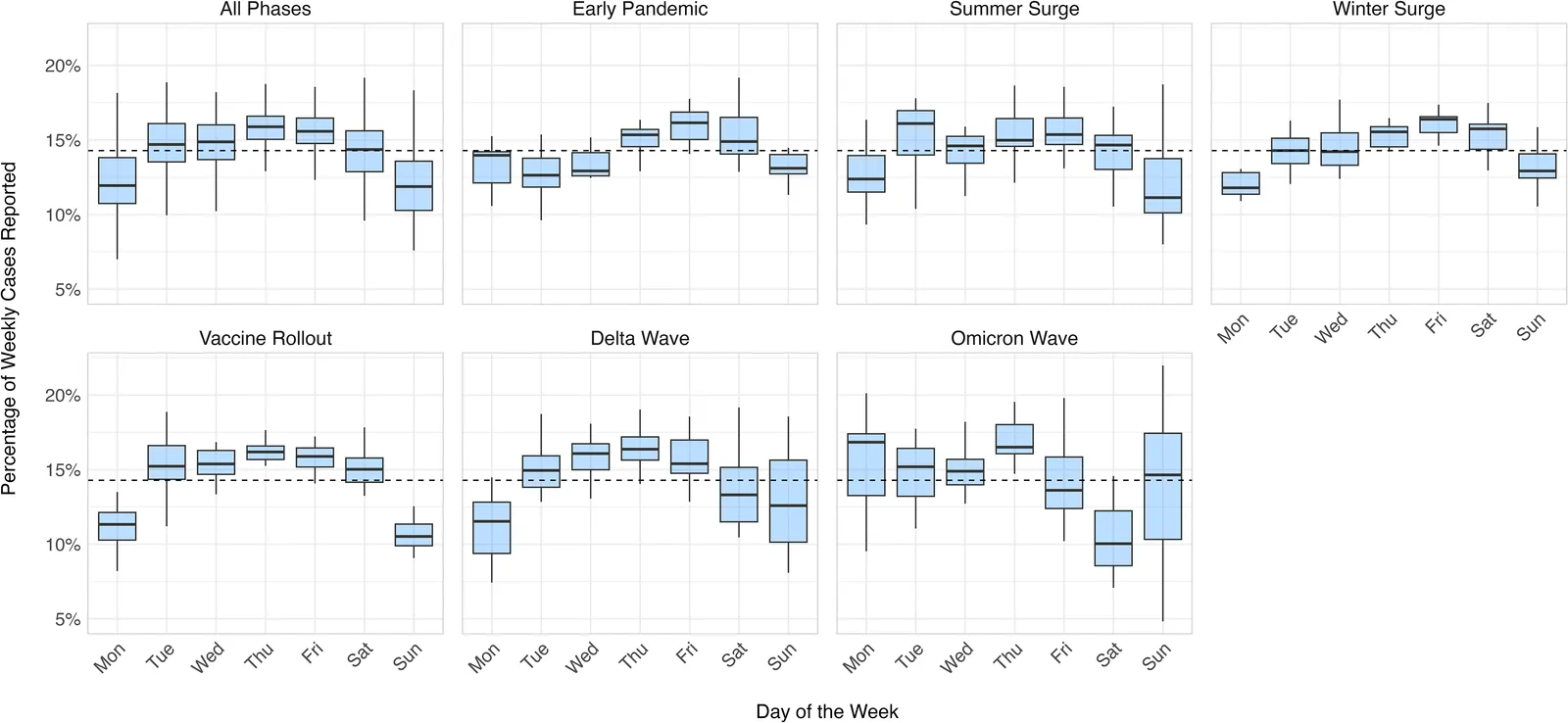
Relative reporting of COVID-19 cases by day of the week for each outbreak phase. Each phase corresponds to those shown in Fig 1. Boxplots show the distribution of the percentage of weekly cases reported by day of the week, across weeks. Boxes represent the interquartile range (IQR), lines show medians, and whiskers extend to the most extreme values within 1.5 × IQR. Dashed lines represent the expected percentage if cases were reported equally on each day.

The day of the week pattern of COVID-19 case reporting also varies by outbreak phase (Fig 3). The Summer Surge, Winter Surge, Vaccine Rollout and Delta Wave phases exhibited a similar overall trend to that observed when aggregating across all phases, with a smaller median percentage of weekly cases reported on Sundays and Mondays, with peaks in reporting typically occurring midweek. The median percentage of cases reported peaked on Thursdays for the Vaccine Rollout and Delta Wave, while reported cases typically peaked on Tuesdays and Fridays for the Summer and Winter Surges, respectively. The Early Pandemic exhibited a different trend, with most cases reported from Thursdays to Saturdays, with lowest reporting on Tuesdays. The Omicron Wave, showed the most irregular pattern, with peaks on Thursdays and lowest reporting on Saturdays. Both the Delta Wave and Omicron Wave showed considerable week-to-week variability in daily reporting patterns, particularly on Sundays and Mondays.

### Forecasting performance by US state

**Fig 4:**
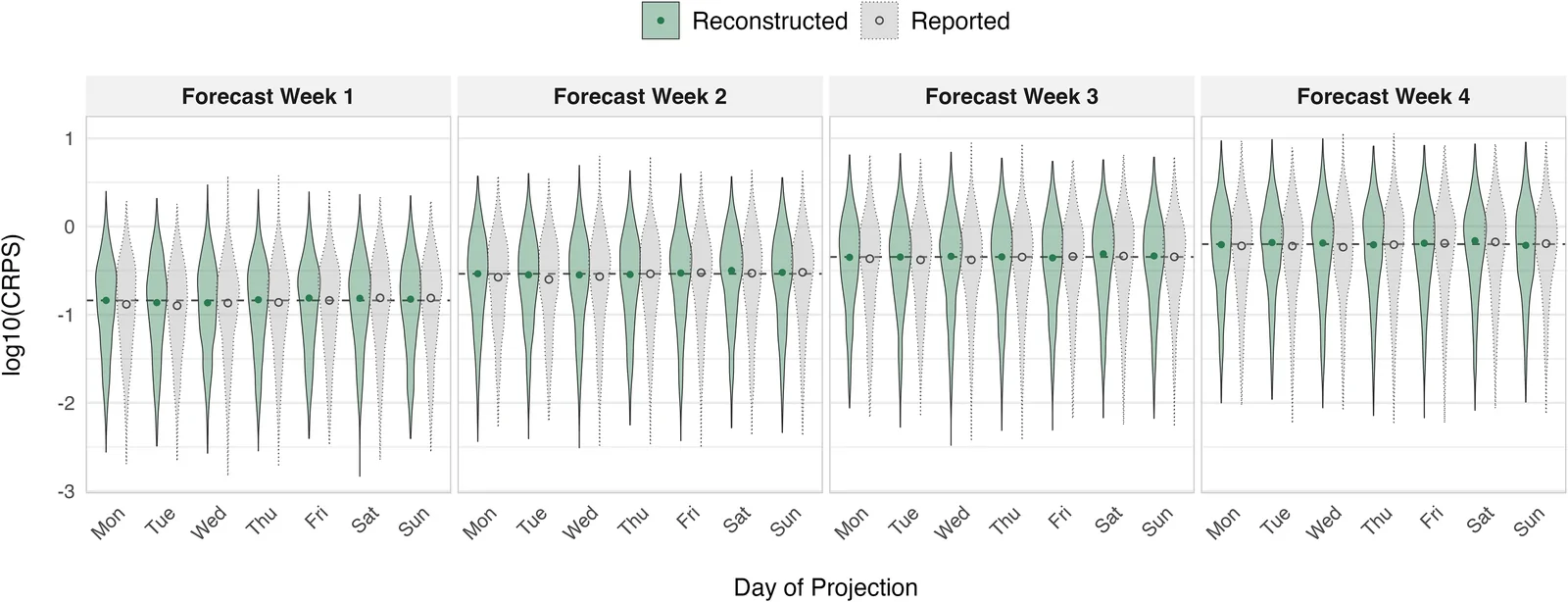
Violin plot showing the distribution of log-transformed (base 10) CRPS values across all included US states, based on the difference in true observed weekly cases and weekly forecasts produced from reconstructed (green) and reported (grey) incidence data. Points denote the median CRPS values and the dashed black lines represent the overall median CRPS score across all included US states in each forecast week. The distribution of CRPS values for each US state individually can be found in S2 Fig.

Averaged across all 13 states included in the analysis, CRPS values showed only marginal differences within each forecast week (Fig 4), irrespective of whether reported or reconstructed incidence were used or the day of the week chosen for projections. As expected, CRPS values progressively increased over the forecast weeks, reflecting reduced performance over longer forecasting horizons.

**Fig 5:**
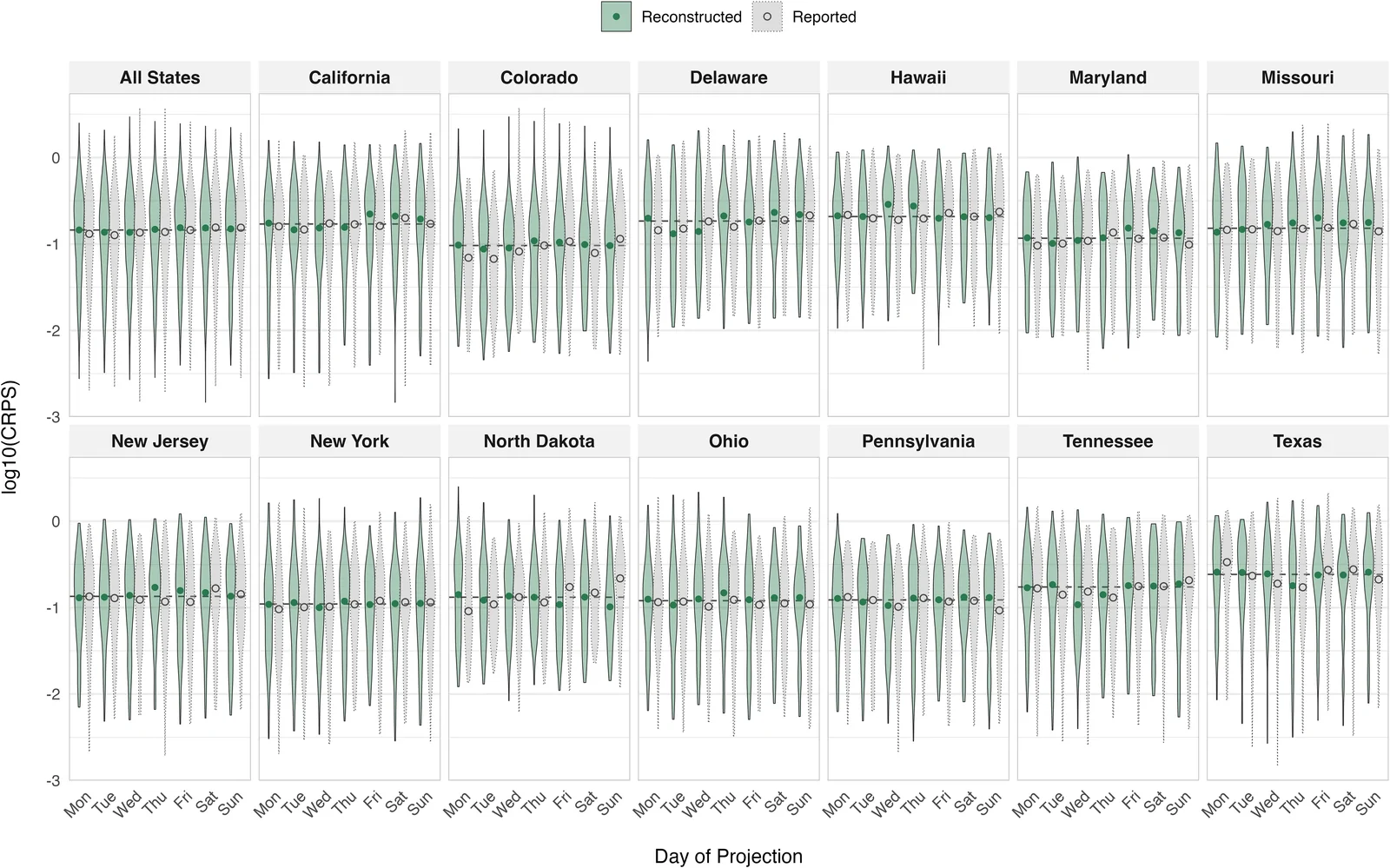
Violin plot showing the distribution of log-transformed (base 10) CRPS values by US state for the first forecast week, based on the difference in true observed weekly cases and weekly forecasts produced from reconstructed (green) and reported (grey) incidence data. Points denote the median CRPS values and the dashed black lines represent the overall median CRPS score for each state in the first forecast week. The “All States” panel shows the distribution of CRPS values across all individual state forecasts. The distribution of CRPS values for forecast weeks 2 to 4 can be found in S2 Fig.

When considered separately, each state follows the same general trend of increasing CRPS over the forecast weeks (S2 Fig). Within each forecast week, CRPS values are typically similar, but for some states there are small differences in performance depending on the projection day. For example, Friday and Sunday forecasts based on reported data appear to perform consistently worse (higher median CRPS values) in North Dakota (Fig 5 and S2 Fig).

### Forecasting performance by outbreak time period

**Fig 6:**
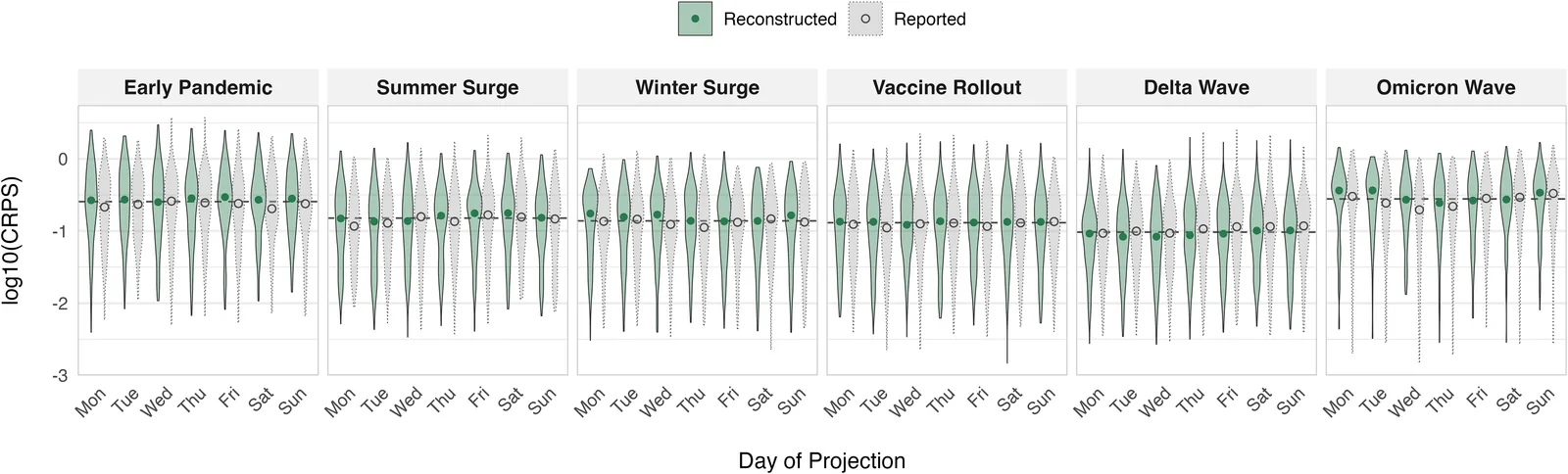
Violin plot showing the distribution of log-transformed (base 10) CRPS values for all included states by outbreak time period in forecast week 1, based on the difference in true observed weekly cases and weekly forecasts produced from reconstructed (green) and reported (grey) incidence data. Outbreak phases correspond to those shown in Fig 1. Points denote the median CRPS values for each of the projection days (last day of past incidence data supplied to the model). The dashed black lines represent the overall median CRPS score for each outbreak phase in the first forecast week. The distribution of CRPS values for forecast weeks 2 to 4 can be found in S3 Fig.

When considering outbreak time periods separately (Fig 6), forecasting performance was worst overall (higher combined median CRPS in each forecast week) during the Early Pandemic (March – May 2020) and the large Omicron Wave (December 2021 – February 2022). During the Early Pandemic, forecasts using reported incidence data perform slightly better, other than Saturdays in the final forecast week (S3 Fig). During the Omicron Wave, Wednesday forecasts using reported data perform best. In the Summer Surge (June – September 2020), Winter Surge (October – December 2020), Vaccine Rollout (January – June 2021) and Delta Wave (July – November 2021), differences between forecasts appear negligible (Fig 6).

## Discussion

Branching process models are especially valuable during the early stages of outbreaks when limited data make it challenging to reliably parameterise more complex models. In this study, we applied the EM algorithm to US COVID-19 case incidence data to demonstrate that reconstructed incidence data can achieve comparable forecasting performance to reported incidence data regardless of the projection day (i.e., the last day of past incidence data supplied to the model), US state, or outbreak time period. The findings suggest that while daily reporting may offer some advantages under specific circumstances, such as during the Early Pandemic, the differences in forecasting accuracy are generally minimal. This highlights the viability of reconstructed data as a practical alternative for supporting accurate forecasting efforts in contexts where setting up and maintaining daily reporting is logistically challenging or resource-intensive.

### Forecasting performance across US states

For all included US states combined, model performance was similar regardless of the projection day or whether daily incidence data were reported or reconstructed (Fig 5). Increasing median CRPS values across forecast weeks (indicating worse performance) highlights the inherent uncertainty in predictions with longer forecasting horizons (Fig 4 and S2 Fig). This pattern is expected, as projections assume that transmissibility remains the same as in the calibration window of past incidence data throughout the forecasting period. Over longer time frames this assumption becomes more unlikely due to factors such as increasing behaviour change and the implementation or relaxation of interventions [1].

When US states were considered independently, there was no clear advantage for the use of either reported or reconstructed incidence data. However, the choice of projection day influenced forecasting performance for some individual states (Fig 5 and S2 Fig). US states recorded cases by the date they were reported, which is delayed from the actual test date, and were lowest on Sundays and Mondays with a relatively even distribution around a peak on Thursdays (Fig 2), which is similar to the trend observed across all US states by the COVID Tracking Project between March 2020 and February 2021 [31]. This is different to patterns observed in the UK, where case counts were recorded by the date of specimen collection for testing and typically exhibited a ‘weekend-effect’ with lower case counts from Friday to Sunday and relatively stable reporting over the rest of the week [9].

Each US state has its own health department and reporting practices, which lead to state-specific differences in reporting patterns (Fig 2). Most US states reported data with a delay of one or two days, causing a corresponding shift in the ‘weekend-effect’ to the beginning of the following week [31]. There was no obvious correlation between state-specific reporting patterns (Fig 2) and the best performing projection days in forecasts. The model calibration window (see methods) was chosen to smooth out reporting patterns in the data and smooth out discontinuities in the reconstructed incidence. These discontinuities occur as an artefact of the constraint in the EM algorithm, which requires that reconstructed incidence sum exactly to the original aggregated case counts [9]. However, this 10-day window would have encompassed either two peaks or two dips in the relative reporting of cases, which could have caused projection day variations in the results. When using reconstructed incidence, calibration windows should always be at least the length of the original data aggregation and longer windows are recommended to help mitigate these discontinuities [9].

Given the differences observed in forecasting performance, multiple projection days could be considered for real-time analysis. It is important to also consider the practical utility of forecasts depending on the specific day of the week they are generated. For example, projections made using incidence data up to Sunday are performed on Mondays, which can guide the week’s response [1]. The timing of incidence data publication will also play a role, as some datasets may be updated daily, while others may only be released once a week, limiting flexibility in when forecasts can be performed for real-time response. Therefore, the choice of projection day should balance practical constraints with potential gains in forecasting performance.

### Forecasting performance across outbreak phases

When considering distinct outbreak phases (Fig 1), forecasts made during the Summer Surge to the Delta Wave (June 2020 to November 2021) showed minimal differences in forecasting performance regardless of the dataset and projection day (Fig 6 and S3 Fig). In contrast, forecasting performance was notably worse in the early phase and the Omicron Wave (Fig 6 and S3 Fig). Several factors likely contributed to this disparity. Reporting patterns differed most noticeably in the Early Pandemic and Omicron Wave (Fig 3). During the early phase, US states had never collected data at the scale the pandemic demanded before, contending with considerable test shortages and longer reporting delays as testing facilities and health departments adjusted to the initial surge in cases [32]. Despite these limitations, forecasts based on daily reported incidence consistently outperformed those using reconstructed incidence, emphasising the importance of prioritising reported daily incidence for forecasting in the early stages of outbreaks.

During the Omicron Wave, healthcare systems were also under immense strain, which could have led to inconsistencies in reporting and therefore worse forecasting performance. Taking into account different projection days and datasets, there were only small differences between them during the Omicron Wave, but reported incidence data with a Wednesday projection day performed best.

### Limitations

While this study provides valuable insights, there are some limitations. Forecasting performance and the reconstruction of incidence data itself can be influenced by high sensitivity to the specified serial interval (or generation time), as even small errors can lead to significant discrepancies in projected outcomes [33]. Given that the method used does not allow for a time-varying SI, the SI chosen for this analysis aimed to provide a reasonable midpoint for the SI throughout the pandemic and was inspired by real-world COVID-19 forecasting efforts [1]. Although applying phase-specific SIs could potentially improve the performance observed in the Early Pandemic or Omicron waves (Fig 6), this limitation is inherent to all renewal equation-based frameworks and is not unique to the reconstruction approach interrogated here [34]. It may also explain the improved performance of forecasts using reported data in the early phase. Future sensitivity analyses could explore whether performance improves with varying SI distributions.

Our approach uses a Poisson likelihood, which assumes that the mean and variance of the incidence data are equal. In practice, incidence data are often overdispersed, which means we may be underestimating the uncertainty of our forecasts, particularly over longer forecasting horizons. Alternative distributions, such as the negative binomial, may be better suited to capturing this overdispersion; however, it can be challenging to quantify the overdispersion parameter across varying outbreak contexts. While future research could explore the effect of alternative likelihood specifications, this would not be expected to change our conclusions regarding the relative performance of forecasts using reported versus reconstructed incidence data.

Additionally, the forecasts are based on a single daily incidence reconstruction. This deterministic approach allows daily incidence to be reconstructed rapidly, which is particularly advantageous for real-time outbreak analysis, but it may lead to an underestimation of uncertainty in our forecasts. However, our analysis demonstrates (S1 Fig) that the reconstructed incidence successfully smooths out administrative noise and captures the underlying epidemiological signal without introducing systematic bias. While alternative reconstruction methods could be considered, the EM algorithm has been shown to outperform approaches such as smoothing splines while maintaining data integrity by ensuring reconstructed counts sum exactly to the original weekly totals [9].

The weekly smoothing of incidence that occurs during reconstruction, combined with the model’s 10-day calibration window, could influence forecasting accuracy during periods of rapid changes in transmission or reporting frequency, such as the Early Pandemic and Omicron Wave. The choice of calibration window is a trade-off between noise reduction and temporal resolution; while we chose a 10-day window for stability and consistency with previous real-world COVID-19 analyses [1], future research could interrogate forecast sensitivity to alternative window lengths to optimise the detection of turning points in the epidemic curve. In terms of the smoothing across days within each aggregation period, the temporal resolution of the reconstruction is ultimately constrained by the reporting frequency of the surveillance data.

It is also important to consider how the reporting rate varies over the course of an outbreak [35]. For instance, if the healthcare system is overwhelmed and does not have the capacity to handle an influx of cases, the proportion of reported cases would likely be lower than during time periods where the healthcare system is under less pressure. If the proportion of cases reported during the forecast period differs to the calibration window, this will lead to bias in comparisons between forecasts and observed weekly counts. Time-varying reporting rates could be considered in future research.

Although our focus has been on weekly reporting of COVID-19 cases in the US, the approach presented here is broadly applicable to other pathogens and various forms of temporally aggregated count data. This approach is particularly relevant for other disease surveillance metrics, such as hospital admissions and mortality data, which are frequently affected by similar administrative reporting delays. The utility of this framework extends even beyond epidemiology to any field or context where granular trends need to be recovered from periodically aggregated time-series data, such as ecology or economics. In the current context, whilst we have shown that forecasting performance is comparable, determining the optimal reporting frequency for surveillance systems should consider the specific characteristics of the pathogen (e.g., generation time), as well as the public health infrastructure and context of the country or region of interest. Ultimately, our findings suggest that when the integrity of the data is maintained through robust reconstruction methods, it is possible to mitigate the limitations of less frequent reporting without compromising the ability to generate reliable forecasts and maintain situational awareness during an outbreak.

## Conclusion

Reconstructed incidence data can support reliable and accurate forecasting when daily reported incidence is not available. While daily reported incidence remains critical during the early stages of outbreaks, the findings emphasise the viability and practicality of using reconstructed incidence for diseases where cases are typically reported over longer timescales or when reporting changes arise during prolonged outbreaks. Balancing the benefits of daily reporting with the flexibility offered by reconstructed data can ensure more resilient forecasting methods in real-time outbreak response.

## Supporting information

S1 Fig Comparison of reconstructed and reported daily COVID-19 case incidence across US states. Top panels for each state display the reconstructed daily incidence (green line) and the reported daily incidence (grey line). Bottom panels show the daily residuals, calculated as the reported minus the reconstructed daily counts. The “All States” panel shows the incidence for all included US states combined. Note that for clarity the y-axes scales are not shared between panels.
(TIF)

S2 Fig Violin plot showing the distribution of log-transformed (base 10) CRPS values by US state, based on the difference in true observed weekly cases and weekly forecasts produced from reconstructed (green) and reported (grey) incidence data. Points denote the median CRPS values and the dashed black lines represent the overall median CRPS score for each state in each forecast week. The “All States” panel shows the distribution of CRPS values across all individual state forecasts for each forecast week.
(TIF)

S3 Fig Violin plot showing the distribution of log-transformed (base 10) CRPS values for all included states by outbreak time period, based on the difference in true observed weekly cases and weekly forecasts produced from reconstructed (green) and reported (grey) incidence data. Outbreak phases correspond to those shown in Fig 1. Points denote the median CRPS values for each of the projection days (last day of past incidence data supplied to the model). The solid black lines represent the overall median CRPS score for each outbreak phase in each forecast week.
(TIF)
